# Gray and White Matter Distribution in Dyslexia: A VBM Study of Superior Temporal Gyrus Asymmetry

**DOI:** 10.1371/journal.pone.0076823

**Published:** 2013-10-01

**Authors:** Marjorie Dole, Fanny Meunier, Michel Hoen

**Affiliations:** 1 Laboratoire de Psychologie et NeuroCognition, CNRS UMR 5105, université Pierre Mendès France, Grenoble, France; 2 L2C2, CNRS UMR 5304, Institut des Sciences Cognitives, Lyon, France; 3 INSERM U1028, Lyon Neuroscience Research Center, Brain Dynamics and Cognition Team, Lyon, France; 4 CNRS UMR 5292, Lyon Neuroscience Research Center, Brain Dynamics and Cognition Team, Lyon, France; 5 Université de Lyon, Université Lyon 1, Lyon, France; University Of Cambridge, United Kingdom

## Abstract

In the present study, we investigated brain morphological signatures of dyslexia by using a voxel-based asymmetry analysis. Dyslexia is a developmental disorder that affects the acquisition of reading and spelling abilities and is associated with a phonological deficit. Speech perception disabilities have been associated with this deficit, particularly when listening conditions are challenging, such as in noisy environments. These deficits are associated with known neurophysiological correlates, such as a reduction in the functional activation or a modification of functional asymmetry in the cortical regions involved in speech processing, such as the bilateral superior temporal areas. These functional deficits have been associated with macroscopic morphological abnormalities, which potentially include a reduction in gray and white matter volumes, combined with modifications of the leftward asymmetry along the perisylvian areas. The purpose of this study was to investigate gray/white matter distribution asymmetries in dyslexic adults using automated image processing derived from the voxel-based morphometry technique. Correlations with speech-in-noise perception abilities were also investigated. The results confirmed the presence of gray matter distribution abnormalities in the superior temporal gyrus (STG) and the superior temporal Sulcus (STS) in individuals with dyslexia. Specifically, the gray matter of adults with dyslexia was symmetrically distributed over one particular region of the STS, the temporal voice area, whereas normal readers showed a clear rightward gray matter asymmetry in this area. We also identified a region in the left posterior STG in which the white matter distribution asymmetry was correlated to speech-in-noise comprehension abilities in dyslexic adults. These results provide further information concerning the morphological alterations observed in dyslexia, revealing the presence of both gray and white matter distribution anomalies and the potential involvement of these defects in speech-in-noise deficits.

## Introduction

Dyslexia is a neurodevelopmental disorder affecting the acquisition of reading and spelling abilities in the absence of other neurological disorders and despite typical intelligence and a favorable socio-educational environment [1]. Although it is a developmental disorder, the difficulties associated with dyslexia are long lasting and remain throughout adulthood [2-4]. It has been suggested that dyslexic children present a deficit in the processing of phonological information, preventing the efficient acquisition of phoneme-to-grapheme conversion rules. This phonological deficit is manifested through reduced performance in tasks that quantify phonological awareness (e.g., rhyme games or phoneme deletion tasks) [4,5], different aspects of verbal memory [6,7], the repetition of complex pseudowords or rare words [4], or rapid automatized naming [4,8]. Associated with this phonological deficiency, speech perception impairments have also been reported in dyslexia. Although difficult to demonstrate in optimal listening situations, these deficits are highly replicable when speech must be perceived in challenging conditions, e.g., when masked by noise [9-13]. Recently, we presented speech in different backgrounds and under different listening configurations, showing that the severity of this deficit was highly dependent on the type of noise presented in the background and the listening configuration tested [14]. In particular, the speech-in-noise comprehension deficit of dyslexic adults was specifically observed when the concurrent sound was speech compared with other speech-derived noises. This observation reinforces the hypothesis of a specific difficulty with the processing of speech information (i.e., phonological information) in dyslexia; however, the cerebral origins mediating this deficit remain largely undetermined.

In this context, different neuroimaging studies have shown an association between behavioral deficits in dyslexia and underlying cerebral dysfunctions and/or morphological anomalies in the cortical regions directly involved in reading or speech processing [15,16] (for a review, see [17]). The cerebral network underlying phonological processing is well known. This network largely overlaps with the more general speech network (for reviews, see [18,19]) and primarily concerns cortical regions along the posterior region of the superior temporal gyrus (STG), Brodmann’s area (BA) 22, with a leftward functional asymmetry outside of the primary and secondary auditory areas (BA41/BA42). This network also contains the posterior region of the inferior frontal gyrus (IFG), BA 44/6 [20]. When dyslexic individuals are compared with normal readers in fMRI experiments, the observed functional differences most often consist of hypo-activations in these brain regions or in regions engaged in other aspects of reading or speech processing, such as the medial and inferior temporal gyri (MTG, ITG), or the left fusiform gyrus, which are all involved in visual word recognition [21-23]. Beyond the magnitude of the cerebral activations, which are decreased in dyslexics, alterations in the inter-hemispheric distribution of functional activation inside the speech-processing network were also reported. Particularly, dyslexics often show decreased activation in the left hemisphere [22-25], which is in some studies even associated with increased activation in the homologous right hemisphere [26]. These observations might reflect the functional hemispheric specialization of language-related networks, which are highly specialized towards the left cortical hemisphere in typical participants, showing more interindividual variability in dyslexics and less asymmetry in general. Consistent with this idea, atypical functional asymmetry patterns have been observed in dyslexics or in children with reading disabilities, at primary stages of auditory processing pathways, such as in the auditory brainstem [27] or the primary auditory cortex [28,29]. Using magnetoencephalography (MEG), Heim et al. [28] reported the atypical asymmetry of the P100m, which is evoked in response to the presentation of a /ba/ syllable in dyslexic children and adolescents. P100m generators were asymmetric in normal readers, but symmetric in dyslexics. Heim and colleagues subsequently confirmed this observation, showing a similar pattern of results for the N100m generators in dyslexic adults [29]. More recently, and in the framework of the asymmetric sampling in time (AST) theory [30,31], evidence of alterations in asymmetric sampling in auditory cortices was reported in dyslexics [32]. In this study, dyslexics showed a reduced entrainment for rapid modulations in the left auditory cortex potentially resulting in the impaired extraction of phonemic cues in the left hemisphere. In addition, the right auditory cortex of dyslexic individuals also demonstrated enhanced entrainment to rapid modulations, suggesting atypical functional inter-hemispheric specialization for slow and fast sampling rates. Moreover, consistent with the idea of a close association between the atypical functional asymmetries of the speech processing network in dyslexia and the development of deficient phonological abilities, several studies have shown that the ability of typical listeners to reconstruct perturbed, e.g., time-reversed speech signals, was positively associated with the functional asymmetry of the peripheral auditory system [33], and dyslexic participants exhibited atypical peripheral asymmetry [27]. Moreover, a functional re-asymmetrization could be stimulated in dyslexics through phonological training, with measurable effects at both the cortical [34] and cochlear levels [27]. The question of the causal association between functional asymmetry inside the speech network and the phonological deficit observed in dyslexia remains unanswered, as the acquisition of literacy skills modifies the development of phonological processes in normal reading children [35]. One approach to potentially understand the origin of functional asymmetrization deficits observed in dyslexia is to associate these deficits with the underlying morphology of the concerned brain regions. Multiple studies have shown that the behavioral and functional changes related to experience-dependent plasticity were also associated with modifications of the underlying cortical morphology, particularly, in the distribution of gray and white matter (see [36] for a review). According to previous anatomical studies, the functional asymmetry abnormalities observed in dyslexia might be associated with underlying morphological anomalies, as suggested through the pioneering research of Galaburda and colleagues [37,38], which suggested the existence of a reduced leftward asymmetry in the planum temporale in reading-impaired individuals, a region involved in speech and auditory processing [39,40]. Although methodological concerns have since challenged these early observations, and subsequent studies using MR imaging have produced mixed results, some of them consistent with the findings of Galaburda [41-44], whereas other studies have not replicated these results [45-48], morphological abnormalities in dyslexia have often been reported. Atypical morphological asymmetry was observed outside the planum temporale proper, including the entire temporal lobe [49], the parietal operculum and Broca’s area [47]. The question of the anomaly of functional and/or morphological asymmetries in dyslexia thus remains controversial, and the origin for this discrepancy might reflect the heterogeneity of the cognitive profiles of the participants included in the different studies. Indeed, a study designed to differentiate brain abnormalities in participants with specific language impairment (SLI, characterized by poor oral language skills, associated with reading deficits) versus dyslexia, suggested that symmetrical brain structures are associated with cognitive profiles, such as SLI, whereas dyslexic individuals have asymmetrical brain structures [48]. This result emphasized the need for a careful examination of the cognitive profiles of the participants and further investigation of the morphological abnormalities in dyslexia. Voxel-based morphometry (VBM), a morphometric technique that enables the separation of gray (GM) and white matter (WM) volumes, is useful for the investigation of morphological abnormalities in dyslexia. Brambati et al. [50] reported reduced GM volumes bilaterally in the planum temporale, fusiform gyrus, and in the left inferior and superior temporal gyri and right middle temporal gyrus. Gray matter volume changes in the left and/or right temporal regions were confirmed through different studies [51-54]. Furthermore, studies associated with WM abnormalities in dyslexia, such as VBM and diffusion tensor imaging (DTI) studies, showed reductions in the volume/density or connectivity along the arcuate fasciculus, including Broca’s area and the temporal cortex [55-59].

The relationship between anatomical asymmetries in temporal regions and the behavioral deficits consistently observed in dyslexia remains unclear, and currently, there are no VBM studies specifically reporting brain asymmetry abnormalities in dyslexic individuals. However, VBM can be efficiently used to derive asymmetry maps of the cortical surface and to quantify the morphological asymmetry observed in the distribution of the GM and WM [60-62]. Thus, the aim of the present study was to compare the asymmetry patterns of the GM and WM volumes along the superior and medial temporal regions in dyslexic adults and normal readers and examine the relationship between these asymmetries and the speech-in-noise deficit associated with dyslexia. Thus, we directly compared the distribution of GM and WM in dyslexic and normal reading individuals and investigated the relationship of these effects with speech-in-noise perception abilities.

## Materials and Methods

### Participants

Fourteen dyslexic participants (6 females, mean age: 23.29 years, S.D.: 6.08) and 14 normal readers (6 females, mean age: 26.07 years, S.D.: 6.19) participated in this study. All subjects were right-handed (scores of ≥70 on the Edinburgh Handedness Inventory [63]), with audiometric pure-tone thresholds of ≤25 dB on a frequency range from 250 to 8000 Hz. Statistical analyses (t-test, all *P*>0.05) confirmed that both groups did not significantly differ in age, nonverbal-IQ or handedness (Table 1). All dyslexic individuals reported a childhood history of reading/spelling disorders, and all but one participant in the normal readers group were also screened for reading, spelling, phonological and verbal short-term memory abilities. The participants reported no history of psychiatric or neurological disorders. In addition, all participants provided written informed consent and were paid for participation. The protocol used in this experiment was approved through a local ethics committee (CPP Sud-Est IV, Lyon; ID RCB: 2008-A00708-47).

**Table 1:** Psychometric evaluation scores in dyslexic and normal reading participants.

**Variable**			**Dyslexics**	**Normal Readers**	**p-value**
Age (years)			23.29 (6.08)	26.07 (6.19)	0.241
Gender (male/female)			8/6	8/6	-
Handedness			84.29 (10.16)	90 (11.09)	0.167
Raven’s (percentile)			49.07 (3.83)	51.69 (4.25)	0.104
Reading age (months)			138.46 (19.47)	168 (4.53)	<0.001*
Reading (words)	Regular	Score (/20)	19.64 (0.63)	20 (0)	0.053
		Time (s)	15.00 (5.07)	9.83 (1.90)	0.003*
	Irregular	Score (/20)	19.50 (0.85)	19.84 (0.55)	0.227
		Time(s)	13.79 (4.26)	10 (2.12)	0.008*
	Pseudowords	Score (/20)	18.07 (1.82)	19.15 (1.21)	0.083
		Time(s)	25.43 (6.87)	13.38 (2.50)	<0.001*
Spelling	Regular		8.50 (1.45)	9.61 (0.50)	0.015*
	Irregular		9.50 (0.65)	9.84 (0.37)	0.106
	Pseudowords		9.21 (1.05)	9.85 (0.37)	0.051
	Sentences	Orthography	9.14 (1.17)	9.85 (0.55)	0.059
		Grammar	7.21 (2.58)	9.77 (0.6)	0.002*
Phonological awareness	Phoneme deletion	Score (/10)	8.50 (1.51)	10 (0)	0.001*
		Time (s)	36.92 (9.60)	24.08 (4.15)	<0.001*
	Acronyms	Score (/10)	8.50 (1.83)	9.46 (0.78)	0.091
		Time (s)	60.77 (18.45)	42.31 (9.01)	0.003*
Visual assessment	Letter sequences	Scores (/20)	19.54 (0.78)	19.75 (0.45)	0.418
		Time (s)	45.39 (8.44)	35.33 (6.85)	0.003*
	Bell’s test (/35)		34.21 (1.05)	33.77 (1.17)	0.307
Oral skills	Word repetition (/16)		16.00(0)	16.00 (0)	-
	Pseudoword repetition (/20)		19.07 (1.21)	19.75 (0.45)	0.078
	R.A.N (s)		16.00 (3.17)	14.15 (2.19)	0.094
Memory span	Forward digit		5.93 (1.00)	6.61 (0.65)	0.046*
	Backward digit		4.93 (0.47)	5.54 (1.20)	0.090
Speech-in-noise	Dichotic		0.98 (0.03)	1.00 (0)	0.092
	Monaural		0.71 (0.10)	0.80 (0.03)	<0.001*
	Spatialized		0.97 (0.03)	0.96 (0.03)	0.509

Significant group differences are indicated in bold and with an asterisk. Values represent the mean score of each group, the standard deviation is indicated in brackets. All dyslexic participants and all but one participant of the normal readers group took part in these tests. Reading age was derived from the test ‘L’alouette’ [65]; other reading skills, phonological abilities, motor oral skills, visual assessment and memory span were evaluated using the ODEDYS test [66]. The speech-in-noise test was an in-house developed test [14]; values for each group indicate mean intelligibility sores (1=100% intelligibility). R.A.N.: rapid automatized naming.

### Psychometric evaluation and speech-in-noise test

The nonverbal IQ was assessed using Raven standard progressive matrices [64]. All participants obtained normal scores above the 50^th^ percentile (see Table 1 in Results section). The reading-age was assessed using the French ‘L’alouette’ reading test [65], and the neuropsychological battery ODEDYS [66] was administered to all participants. This battery evaluated reading and spelling, metaphonology (acronyms and phoneme deletion tests), verbal short-term memory, visual attention, and rapid automatized naming.

The participants also performed a speech-in-noise intelligibility test, involving the perception of words presented in background babble-noise according to different listening configurations. The materials and procedures of this test have been previously described [14]. The stimuli included 60 disyllabic words embedded in a 5-s background of 4-talkers babble. Target words were selected in an intermediate range of lexical frequencies (0.13–338.19 occurrence per million (opm), average: 16.82 opm, S.D.: 43.74). The number of phonemes and word frequencies were counterbalanced between the conditions. The background babble-noise comprised 4 individual voices recorded in a soundproof room while reading extracted passages of a French book. The individual recordings were modified according to the following protocol: i) removal of silences and pauses of more than 1 s; ii) deletion of sentences containing pronunciation errors, exaggerated prosody or proper nouns; iii) removal of low-amplitude background noise using noise reduction optimized for speech signals (CoolEdit Pro© 1.1 – Dynamic Range Processing – preset Vocal limiter); iv) intensity calibration in dB-A and normalization of each source at 70 dB-A; and v) final mixing of the 4 sources. Three listening configurations were used: Dichotic, Monaural and Spatialized. In the Dichotic configuration, the target words were presented in one ear and babble was presented in the other ear at the same intensity. In the Monaural configuration, the target speech and babble background were presented in one ear only, on the right side, at a signal-to-noise ratio of 0 dB. In the Spatialized configuration, the target speech and babble background were presented in both ears but with an interaural level difference (ILD) for the background of 10 dB, thus mimicking a listening situation in which the target and background are slightly separated in space. Stimuli were presented using headphones (Sennheiser HD 25 SP) at a comfortable hearing level. The participants were asked to repeat the target words. Intelligibility scores were obtained after calculating the proportion of words correctly repeated. A two-way ANOVA was performed, with Group (Normal Readers and Dyslexics) as the between-subject factor and Configuration (Dichotic, Monaural, and Spatialized) as the within-subject factor. Statistical tests were performed at a *P*<0.05 threshold and a post-hoc Bonferroni test was used to investigate specific differences.

### Image acquisition

The MRI acquisition was performed at La Timone Hospital (Marseilles, France) using a 3.0T Brucker Medspec 30/80 AVANCE scanner. One 3D structural image was acquired for each subject, using a T1-weighted MPRAGE sequence: TR: 9.4 ms, TE: 4.42 ms, pulse angle: 30°, field of view: 256 x 256 x 180 mm, matrix: 256 x 256 x 180, and voxel size: 1x1x1 mm^3^.

### Image processing, segmentation and asymmetry analysis

To evaluate the morphological differences between the dyslexic and typical participants, the structural T1 images were used to perform a voxel-wise analysis of the tissue asymmetry, directly derived from the voxel-based morphometry (VBM) approach [67], involving the creation of GM/WM asymmetry maps and performance of between-group comparisons using these maps. The analysis was performed using the VBM8 toolbox (Gaser, http://dbm.neuro.uni-jena.de/vbm), which employs the unified segmentation approach [68] implemented in SPM8 (Wellcome Department of Imaging Neuroscience, http://www.fil.ion.ucl.ac.uk/spm/software/spm8/). This method involves a multiple-step procedure that alternates tissue classification, bias correction and normalization. This toolbox extends the unified segmentation using a maximum a posteriori (MAP) technique [69] and a Partial Volume Estimation (PVE) to account for partial volume effects [70]. The VBM8 toolbox also controls the quality of the procedure by calculating the covariance between the resulting images to confirm the homogeneity of the variance and identify potential outliers.

The T1 images were bias-corrected and segmented into gray matter, white matter and cerebro-spinal fluid maps based on a set of symmetrical a priori Tissue Probability Maps provided in the VBM8 toolbox. The GM and WM maps underwent a spatial-normalization procedure, targeting the standard MNI T1 template. The standard SPM spatial normalization procedure was performed using a 12-parameter affine linear transformation and non-linear warping, by deselecting the DARTEL option in VBM8 [71]. Jacobian modulation was applied to preserve the local GM and WM values, and the voxel values were multiplied by the non-linear components of the registration to account for individual brain size variations. We flipped the GM and WM modulated images along the horizontal plane (x-axis) and applied the formula (original - flipped)/0.5*(original + flipped) using previously described procedures to generate GM and WM asymmetry maps [60-62]. The asymmetry maps were smoothed using a Gaussian kernel of 10 mm (FWHM).

### Statistical analyses

Statistical analysis was performed using the General Linear Model (GLM) [72], implemented in SPM8, according to a two-step procedure: first, whole-brain differences in the GM and WM distribution were separately investigated from the specific tissue maps across each group of participants, using a one-sample t-test, including age and gender as co-variables of non-interest. The cluster-level threshold was set at *P*<0.05 (FWE-corrected), with a voxel-level threshold set at *P*<0.001 uncorrected. A correction for the non-stationarity of smoothness was also applied, allowing cluster-level statistics in VBM data. The significant differences displayed on the right side of the obtained maps represented a rightward morphological asymmetry. The SPM extension Anatomical Automatic Labeling (AAL) [73], and when required, Talairach Daemon [74,75] were used to localize the effects. Second, whole brain group differences in the GM/WM asymmetry were investigated using a two-sample t-test with age and gender as co-variables of non-interest. The statistical threshold was also set at P<0.05 (FWE-corrected at the cluster level). To better characterize the obtained differences in GM/WM asymmetry, we used the GM/WM modulated images obtained after the segmentation/normalization procedure, which were smoothed (10mm FWHM), to extract GM/WM volumes at the location of the significant asymmetry clusters. This allowed to better understand the origins of the asymmetry differences.

For further specific inquires (correlations), the analyses were performed in two symmetric regions of interest (ROI) covering the middle and superior parts of the temporal lobe. These regions were defined using the Marsbar Toolbox (http://marsbar.sourceforge.net/) [76]. In this toolbox, the left and right Heschl’s, superior temporal and middle temporal gyri were selected from the AAL database and combined to form two large regions (the left and right hemispheres), which covered the superior and middle parts of the temporal cortex. Symmetric ROIs were generated after flipping each region along the x-axis, and a mean image from the original and flipped images was subsequently created for each side (Figure S1). In each pre-defined ROI, a small volume correction (SVC) was applied, with the statistical threshold set at *P*<0.05 (cluster level - FWE-corrected). A correlation analysis between the morphological observations and performance on the speech-in-noise intelligibility test was performed. In our speech-in-noise test, only the Monaural condition was associated to significant interindividual variability inside both groups and to a significant difference between groups, the two other conditions leading to roof effects. Therefore, we did not add the results from the other conditions to this model. In each group, the speech-in-noise intelligibility scores in the Monaural configuration were added in the model as a covariable to investigate the voxel-by-voxel correlation in the pre-defined ROIs.

## Results

### Psychometric evaluation

Compared with the normal reading group, the dyslexic participants showed a clear profile of reading impairment, conforming to the symptomatology of dyslexia (Table 1). This profile comprised a significantly lower reading age (Dyslexics: mean age: 138.46 months, S.D.: 19.47 *vs.* Normal Readers: mean age: 168 months, S.D.: 4.53; *P*<0.001), associated with a normal nonverbal IQ in both groups, and no group differences (Dyslexics: average percentile: 49.07, S.D.: 3.83 *vs.* Normal Readers: average percentile: 51.69, S.D.: 4.25; *P*=0.10). The literacy skills of dyslexic individuals were characterized by longer reading times for regular words (*P*<0.005), irregular words (*P*<0.01) and pseudowords (*P*<0.001). The reading accuracy showed a tendency towards a significant difference for regular words (*P*=0.05) and pseudowords (*P*=0.08), not for irregular words (*P*=0.23). The phonological skills were also affected in participants with dyslexia, with lower performances and speeds in the phoneme deletion task (respectively, *P*<0.005 and *P*<0.001) and lower speeds for acronyms (*P*<0.005). Dyslexics also showed impaired verbal short-term memory, with a significant deficit in the forward digit span (*P*<0.05). Moreover, dyslexic participants exhibited normal oral motor skills, with no significant deficit in word repetition or rapid automatized naming (both: *P*>0.05) and a difference in pseudoword repetition accuracy showing a tendency towards significance (*P*=0.08).

The results of the speech-in-noise intelligibility test (see Table 1) confirmed our previous observation of a speech-in-noise perception deficit in dyslexic adults, which was significant only in the most difficult listening situation, Monaural, whereas no significant group difference was observed when comparing the Dichotic or Spatialized conditions [14]. The statistical analyses revealed a significant Group x Configuration interaction (*F*(2,52)=8.60, *P*<0.001), highlighting the presence of this deficit in the Monaural configuration (Normal Readers: average intelligibility rate: 0.80, S.D.= 0.03*vs*. Dyslexics: 0.70, S.D.= 0.1; *P*<0.001) and the absence of this deficit in the Dichotic and Spatialized configurations (both *P*>0.05).

### Voxel-wise analysis of asymmetry - Gray Matter

**Figure 1:**
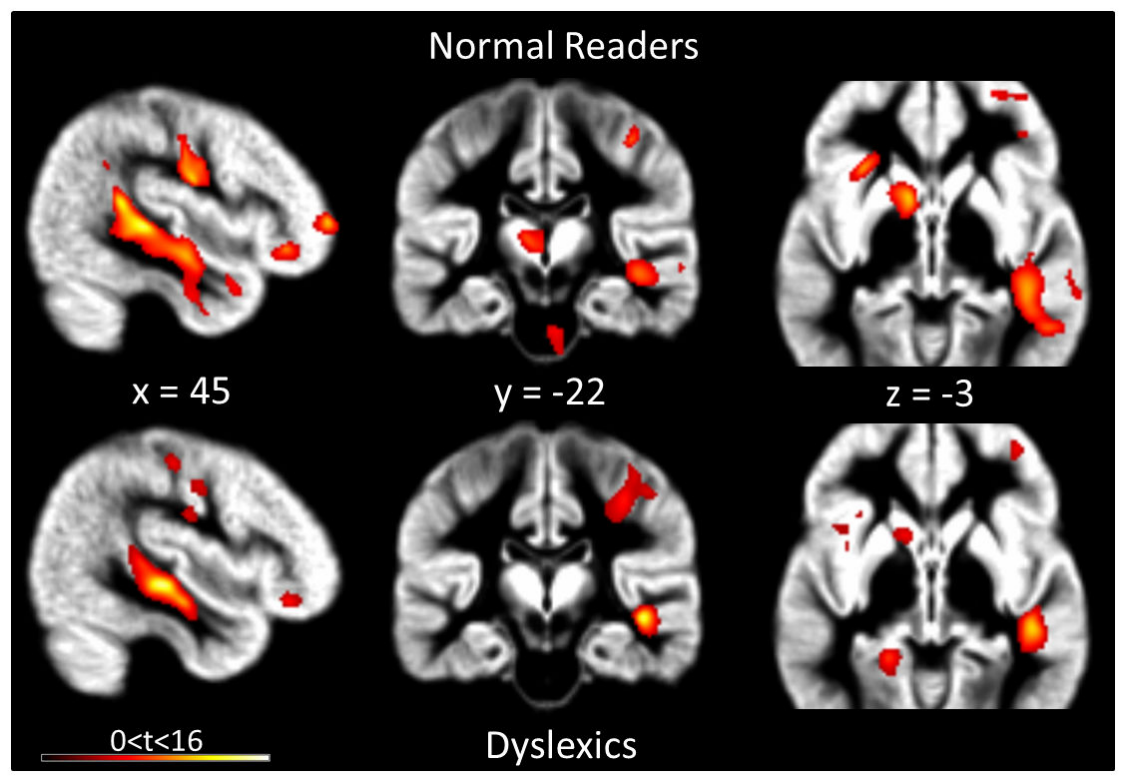
Gray matter asymmetry and whole-brain analysis. The images show cerebral regions with significant (cluster-level FWE-corrected, *P*<0.05) GM distribution asymmetry in normal-readers (upper row) and dyslexic participants (lower row). Significant clusters are displayed on the mean GM image of the whole sample of participants.

In samples from both groups, different cerebral regions were identified as exhibiting a GM distribution asymmetry. Table 2 and Figure 1 show the results of the whole-brain group-specific GM asymmetry analysis.

**Table 2:** Gray matter asymmetries.

	**AAL Region**	**Cluster Size** (voxels)	**Corr. p-value** (clust. level)	**Corr. p-value** (vox. level)	**T max**	**Peak location MNI coordinates**		
						**x**	**y**	**z**
**Normal Readers**								
**Left Hem.**	**Inferior Temporal Gyrus**	**2235**	**<.001**	**0.047**	**11.49**	**-53**	**-37**	**-30**
	**Pallidum**	**2012**	**<.001**	**0.031**	**12.08**	**-12**	**9**	**1**
	Temporal Pole	657	0.008	0.863	6.61	-18	12	-32
	Thalamus	504	0.017	0.793	6.87	-11	-28	6
**Right Hem.**	**STG / MTG**	**4038**	**<.001**	**0.028**	**12.19**	**44**	**-34**	**1**
	**Cerebellum**	**3178**	**<.001**	**0.053**	**11.29**	**12**	**-54**	**-60**
	Medial Cingulate	1292	<.001	0.531	7.71	12	21	36
	**Insula**	**1076**	**<.001**	**0.01**	**13.52**	**38**	**-6**	**24**
	Inferior Frontal Gyrus	950	<.001	0.072	10.79	41	35	-9
	Cuneus	447	<.001	0.26	8.85	9	-84	28
	Medial Frontal Gyrus	356	<.001	0.187	9.35	44	53	3
	Parahippocampal Gyrus	266	0.019	0.263	8.84	6	-18	-30
	Precentral Gyrus	171	0.014	0.675	7.25	33	-19	69
**Dyslexics**								
**Left Hem.**	**Inferior Temporal Gyrus**	**3865**	**<.001**	**0.031**	**12.09**	**-56**	**-42**	**-30**
	**Putamen**	**1959**	**<.001**	**0.178**	**9.4**	**-20**	**9**	**7**
	Medial Cingulate	292	0.009	0.921	6.34	-8	-43	39
	Parahippocampal Gyrus	219	0.002	0.547	7.64	-20	-42	-5
	STG (posterior)	214	0.03	0.115	10.06	-69	-42	13
**Right Hem.**	**STG / MTG**	**1481**	**<.001**	**0.002**	**15.86**	**44**	**-25**	**-6**
	Cerebellum	1750	<.001	0.577	7.54	5	-45	-35
	Inferior Frontal Gyrus	878	<.001	0.052	11.28	32	27	-12
	**Medial Frontal Gyrus**	**573**	**<.001**	**0.047**	**11.46**	**30**	**8**	**39**
	Postcentral Gyrus	500	0.001	0.929	6.29	35	-22	48
	Postcentral Gyrus	488	<.001	0.772	6.93	38	-7	34
	Anterior Cingulate	369	0.041	0.795	6.85	6	42	15

Detailed results from the whole-brain analysis in both participants’ groups. Statistical thresholds set at *P*<0.05 FWE corrected at the cluster level. Results are ranked depending on cluster extent. Asymmetry differences also significant (*P*<0.05 - FWE corrected) at the voxel-level are marked bold.

Regions showing significant leftward asymmetry involved a large cluster centered on the inferior temporal gyrus (left ITG) and extending downwards along the lateral bank of the left cerebellar hemisphere (Normal Readers: k = 2235 voxels, peak at MNI: [-53, -37, -30]; Dyslexics: k = 3865 voxels, peak at MNI: [-56, -42, -30]). A second major cluster comprised the left basal ganglia, with peaks centered on the globus pallidus in normal readers and on the putamen in dyslexic participants (Normal Readers: k = 2012 voxels, peak at MNI: [-12, 9, 1]; Dyslexics: k = 1959 voxels, peak at MNI: [-20, 9, 7]). One significant cluster was present only in the dyslexic group, centered on the left superior temporal gyrus (k = 214 voxels, peak at MNI: [-69, -42, 13]), corresponding to the posterior region of the STG (BA 22). The direct group comparison, however, showed that this difference was not significant (see below).

Regions showing a rightward morphological GM asymmetry revealed a large cluster at the frontier of the right STG and MTG, developing along the right superior temporal sulcus (STS) (Normal Readers: k = 4038 voxels, peak at MNI: [44, -34, 1]; Dyslexics: k = 1481 voxels, peak at MNI: [44, -25, -6]). A second rightward asymmetric cluster was centered on the right cerebellum (Normal Readers: k = 3178 voxels, peak at MNI: [12, -54, -60]; Dyslexics: k = 1750 voxels, peak at MNI: [5, -45, -35]). Moreover, a large cluster was also observed in the right IFG, BA47 (Normal Readers: k = 950 voxels, peak at MNI: [41, 35, -9]; Dyslexics: k = 878 voxels, peak at MNI: [32, 27, -12]). Complete GM asymmetry observations are described in Table 2.

**Figure 2:**
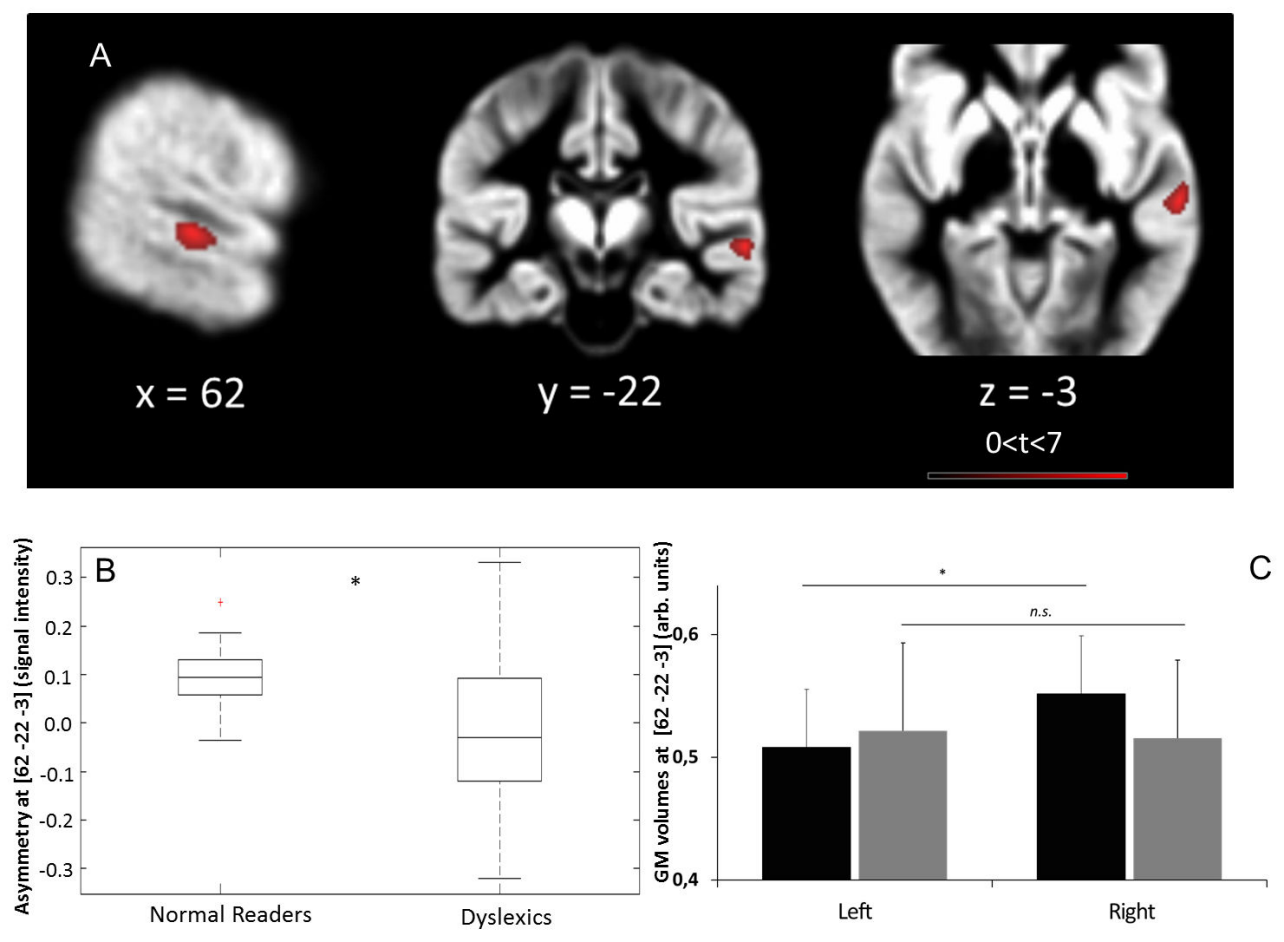
The results from the group difference analysis for gray matter asymmetry. A: significant group difference, suggesting that dyslexics show reduced GM asymmetry at [62, -22, -3]. B: GM asymmetry values (raw signal intensity values expressed in arbitrary units) at [62 -22 -3] in normal readers and dyslexics, illustrating the interindividual variability of GM distribution at this location. C: GM volumes (raw signal intensity values expressed in arbitrary units) at [62 -22 -3] and [-62-22 -3] in normal readers (black) and dyslexics (gray), showing a significant GM asymmetry at that location only for normal readers.

The direct between-group comparison performed on the whole-brain data, revealed that dyslexic participants showed a significant reduction of rightward asymmetry in the STG/STS/MTG cluster compared with normal readers (*P*<0.05 cluster-level FWE-corrected; k= 172 voxels; T=7.58). The peak of this asymmetry difference was located at MNI [62, -22, -3] (see Figure 2A), corresponding to Talairach’s STG BA22, at the interface between STG BA22 and MTG BA21, the STS. This significant difference was associated with an increase in the variability of asymmetry of GM distribution in that location in the dyslexic group (*P*=0.05 on the Levene test on the homogeneity of variance; Figure 2B). To better characterize this difference in GM asymmetry and clarify its origin, we extracted the GM volumes at the peak location on both sides [+/- 62, -22, -3] and in both groups (Figure 2C). A 2-way ANOVA (Group, Hemisphere) confirmed the presence of a significant Group x Hemisphere interaction (*P*<0.05), and a post-hoc LSD test confirmed that normal readers showed increased GM volumes over the right STS compared with the left site (*P*<0.01), while dyslexic participants did not show this difference (*P*=0.68).

In summary, amongst brain regions showing a GM asymmetry in both groups, we identified a large cluster corresponding to a rightward STS asymmetry. When comparing normal readers to dyslexics, dyslexics showed reduced GM asymmetry in this region, and normal readers showed significant asymmetry due to increased GM on the right side.

### Voxel-wise analysis of asymmetry - White Matter

**Figure 3:**
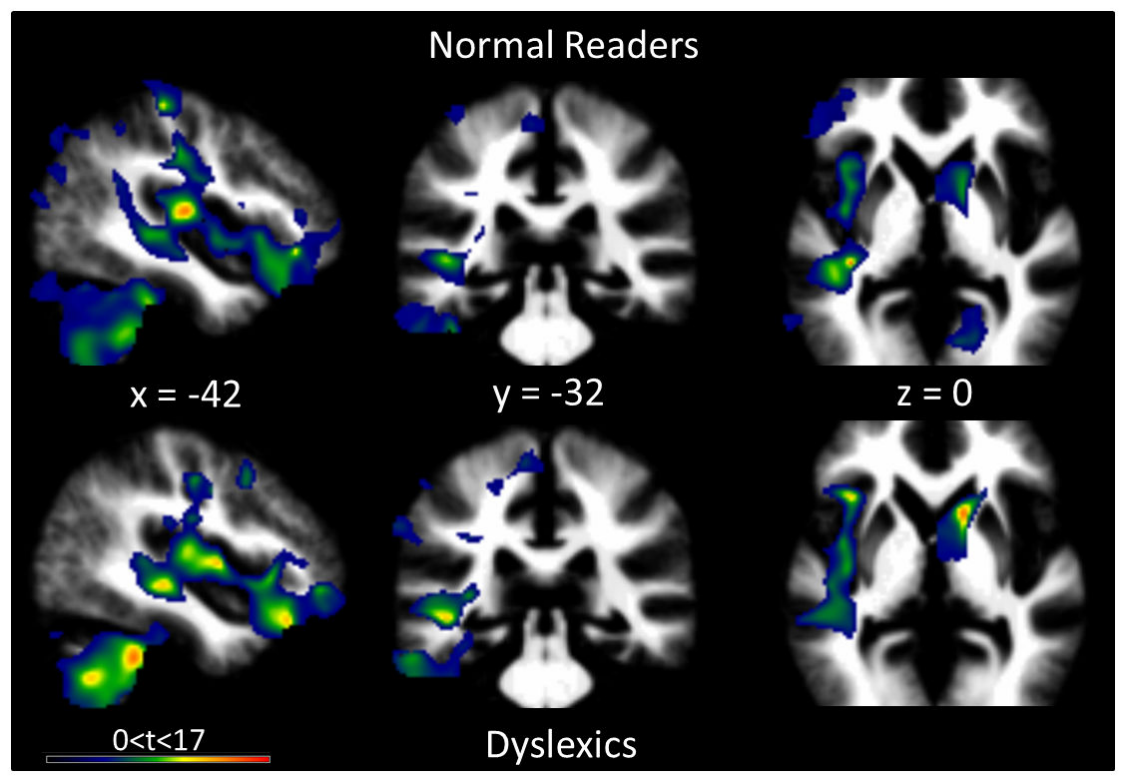
White matter asymmetry, whole-brain analysis. The images show cerebral regions with a significant (cluster-level FWE-corrected, *P*<0.05) WM distribution asymmetry in the normal readers (upper row) and dyslexic participants (lower row). The significant clusters are displayed on the mean WM image of the whole sample of participants.

Whole-brain WM asymmetries were also separately characterized in normal readers and dyslexic participants (see Figure 3 and Table 3).

**Table 3:** White matter asymmetries.

	**AAL Region**	**Cluster Size** (voxels)	**Corr. p-value**(clust. level)	**Corr. p-value**(vox. level)	**T max**	**Peak location MNI coordinates**		
						**x**	**y**	**z**
**Normal Readers**								
**Left Hem.**	**Heschl/STG/IFG**	**18343**	**<.001**	**0.001**	**17.14**	**-50**	**-13**	**10**
	**Cerebellum / ITG**	**16930**	**<.001**	**0.018**	**12.73**	**-38**	**-40**	**-48**
	Medial Occipital Gyrus	2789	<.001	0.054	11.02	-18	-88	15
	Precuneus	466	0.003	0.745	6.87	-2	-67	46
	Paracentral Lobule	274	0.007	0.744	6.87	-8	-36	63
	Precuneus	106	0.018	0.863	6.46	-11	-55	52
**Right Hem.**	Pallidum	3569	<.001	0.181	9.21	12	6	-2
	Medial Frontal Gyrus	841	0.001	0.664	7.12	6	47	-11
	Lingual Gyrus	911	<.001	0.263	8.66	18	-49	-3
	Anterior Cingulate	239	0.041	0.989	5.6	3	50	9
	Calcarine Sulcus	174	0.043	0.617	7.27	33	-54	15
	Putamen	165	0.002	0.035	11.74	27	14	13
**Dyslexics**								
**Left Hem.**	**IFG / Left MTG**	**14983**	**<.001**	**0.001**	**17.47**	**-35**	**23**	**-20**
	**Cerebellum**	**13600**	**<.001**	**0.002**	**15.62**	**-44**	**-40**	**-39**
	Cuneus	238	0.005	0.54	7.49	-18	-64	21
	IFG	218	0.005	0.887	6.34	-50	24	27
	Inferior Parietal	212	0.004	0.487	7.67	-53	-37	55
	MTG	413	0.031	0.981	5.71	-54	-69	12
	Precentral Gyrus	625	<.001	0.257	8.66	-39	11	46
	Superior Occipital Gyrus	75	0.015	0.646	7.15	-15	-87	18
**Right Hem.**	**Caudate**	**3720**	**<.001**	**0.003**	**15.19**	**15**	**14**	**3**
	Superior Frontal Gyrus	391	0.001	0.528	7.53	8	63	9
	Anterior Cingulate	242	0.028	0.909	6.24	2	38	18

Detailed results from the whole-brain analysis in both participants’ groups. Statistical thresholds set at *P*<0.05 FWE corrected at the cluster level. Results are ranked depending on cluster extent. Asymmetry differences also significant (*P*<0.05 - FWE corrected) at the voxel-level are marked bold.

In both groups, the WM was primarily leftward lateralized, with a principal cluster observed in normal readers and dyslexics following the unfolding of the arcuate fasciculus, with a pronounced leftward asymmetry (Normal Readers: k = 18343 voxels; Dyslexics: k = 14983 voxels). Peak locations were less informative given the spatial extent of the cluster, and the details are provided in Table 3. This major leftward asymmetric WM cluster was accompanied by a second large cluster covering the left cerebellar hemisphere and extending into the left inferior temporal gyrus in normal readers and dyslexic participants (Normal Readers: k = 16930 voxels, peak at MNI: [-38, -40, -48]. Dyslexics: k = 13600 voxels, peak at MNI: [-44, -40, -39]).

Regions showing a rightward WM asymmetry were less numerous and concerned a smaller cortical volume but were located in the basal ganglia (Normal Readers: k = 3569 voxels, peak at MNI: [12, 6, -2], globus pallidus; Dyslexics: k = 3720 voxels, peak at MNI: 15, 14, 3] caudate nucleus) and frontal lobe (Normal Readers: k = 841 voxels, peak at MNI: [6, 47, -11], medial frontal gyrus; Dyslexics: k = 391 voxels, peak at MNI [8,9,63]: superior frontal gyrus). Other rightward WM asymmetric clusters comprised the calcarine sulcus in normal readers and the anterior cingulate cortex in dyslexics.

Between-group differences in the WM asymmetry were investigated using a two-sample t-test at the whole-brain level using the same thresholding parameters (FWE-corrected at the cluster level *P*<0.05). This analysis, however, revealed no significant group difference. Performing the same analysis in the bilateral STG ROIs, using small volume correction, did not generate any significant results.

### Correlation between asymmetry and speech-in-noise

**Figure 4:**
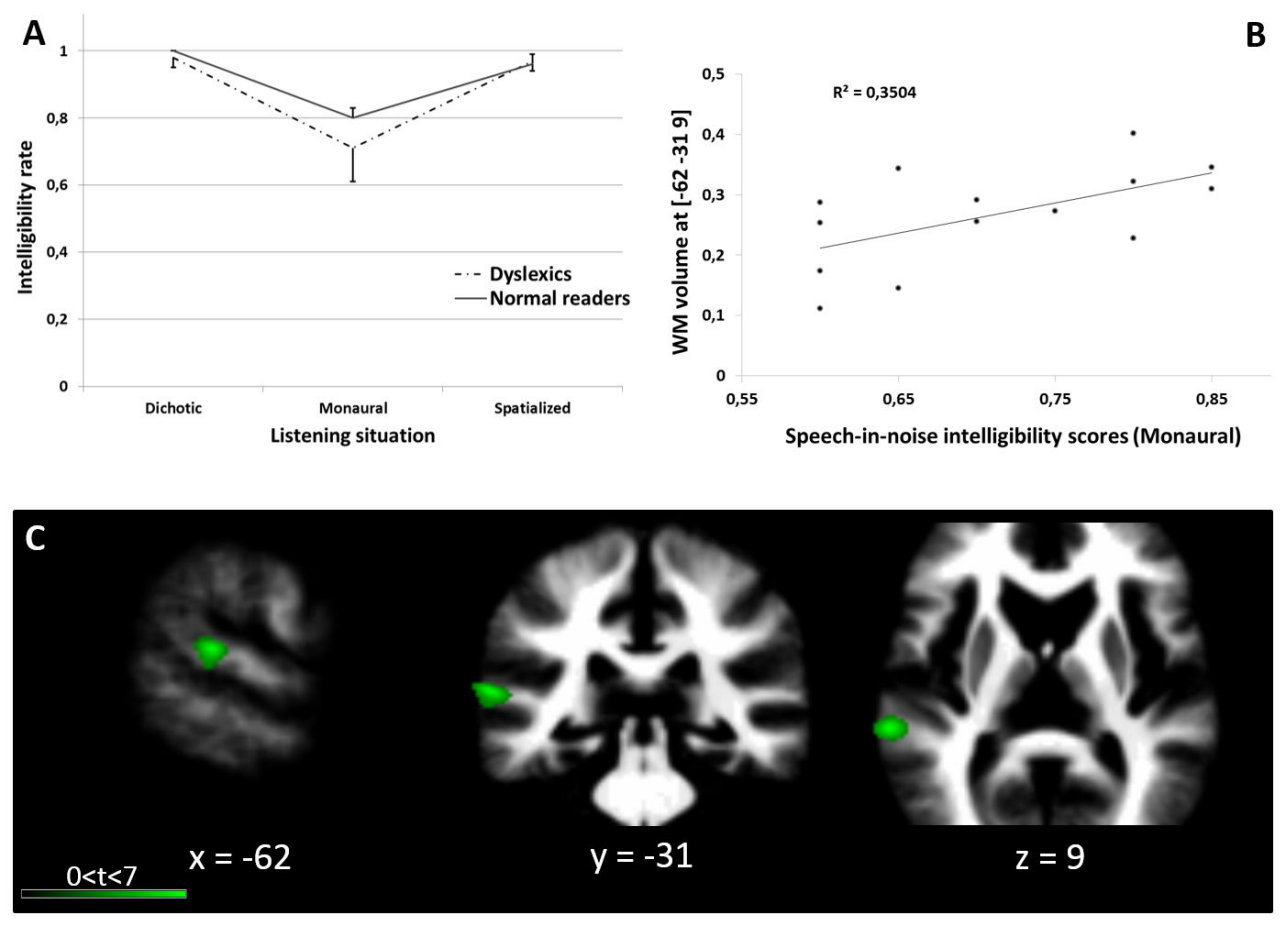
The results of the correlation analysis between the speech-in-noise intelligibility scores in the Monaural configuration and WM asymmetry in dyslexic participants. A: behavioral results of the speech-in-noise comprehension test. The intelligibility of the individual words presented in a 4-talkers babble noise in three listening conditions (Dichotic, Monaural, Spatialized). B: Correlation between the speech-in-noise comprehension scores and WM asymmetry at [-62, -31, 9] for dyslexic participants. C: Visualization of the significant cluster, reflecting the correlation between WM volume and speech in noise scores centered at [-62, -31, 9] in dyslexic participants only.

We performed an analysis of the correlation between the GM and WM asymmetry and the intelligibility scores in the Monaural configuration, associated with significant deficit in dyslexics. Because correlational analyses may yield rather large false-positive rates, we restricted the search of significant correlations to the inside of symmetric superior temporal ROIs (Figure S1). This analysis revealed a significant positive correlation between the intelligibility scores in the Monaural configuration and the leftward WM asymmetry in the posterior STG of dyslexic individuals (Figure 4 A,B,C). Dyslexics: k = 186 voxels, peak at MNI: [-62, -31, 9], *P*<0.05 FWE corrected both at the cluster- and the voxel-level, SVC. No significant correlation between the GM asymmetry and speech-in-noise intelligibility measures was observed.

## Discussion

This study was designed to characterize and compare the inter-hemispheric cerebral asymmetries of gray and white matter distribution in dyslexic adults and normal readers and establish relationships between these morphological characteristics and the speech-in-noise processing deficit observed in dyslexia. To achieve this aim, we explored the distribution of the different types of brain tissue using a VBM-based asymmetry analysis. Correlations with the speech-in-noise intelligibility measures were also performed to examine the structure/function relationships in both groups.

Asymmetry analyses in the WM primarily identified a leftward asymmetry in the arcuate fasciculus, a well-characterized group of WM bundles involved in the specialization of language and speech in the left-hemisphere of the human brain. No group difference between dyslexics and normal readers emerged; therefore, we will not further discuss this aspect. The results of the GM asymmetry analysis demonstrated a significant rightward GM asymmetry involving a large cluster centered at the border between STG BA 22 and MTG BA 21 in both groups of participants, initiating along the superior temporal sulcus (STS) at the caudal extremity and extending forward to approximately two-thirds of the length of the temporal lobe. This observation is consistent with previous morphological asymmetry studies that characterized this GM asymmetry [77,78]. Interestingly, in the present study, this morphological asymmetry was associated with a significant group difference: dyslexic participants showed reduced GM asymmetry in an area located at MNI [62, -22, -3], at about mid-length of the right-STS. The extraction of the GM volumes at this location revealed that, in contrast to normal readers who exhibited significantly more GM on the right compared with the left side, dyslexics did not show this asymmetry. This result is consistent with former observations of reduced functional asymmetry in dyslexia, particularly in regions associated with auditory and speech processing [28,29,32]. Moreover, functional abnormalities have been observed in dyslexic adults in the right STS during word processing [79]. This rightward mid-STS asymmetry could directly reflect the functional specialization of this region for the processing of voice information in humans [80-82]. The locus in which we observed rightward asymmetry was termed the Temporal Voice Area (TVA), described by Grossman et al. [83] and Charest et al. [81]. This area is a counterexample of a cortical region with a potentially important role in speech perception and a clear rightward functional asymmetry [80], potentially resulting from an underlying rightward morphological asymmetry in the GM [84]. This region is clearly sensitive to human voice compared with other complex but scrambled sounds, without the harmonic structure of voicing [85]. Specifically, the anterior to mid STS/STG has been previously linked to processes associated with recognizing and directing attention toward voice characteristics when hearing speech [86]. Furthermore, this region might also be involved in the processing of competing auditory sources [87] and could therefore play a role in the processing of masked speech [88]. The right STS might play an important role in the cortical network during the cocktail-party effect, particularly, during speech-in-speech comprehension. In this situation, the streaming of individual voices, based on the processing of fundamental frequencies, i.e., f0 characteristics, is key to successfully maintaining intelligibility in an environment where multiple voices are present together [89,90]. Indeed, this listening situation constitutes a three-fold challenge, involving the integration of linguistic, spatial localization and auditory object identification processes. Consistently, Altmann et al. showed using different animal vocalizations that could change either the nature or the spatial location of the source, that the right STS was highly sensitive to changes in the identity of the auditory object represented and less sensitive to changes in spatial position [91,92]. Therefore, the right STS region is specifically engaged in the recognition and identification of complex auditory objects belonging to different semantic categories [93,94]. The right STS would therefore be responsible for the object-fold of the speech-in-speech situation, identifying, representing and tracking specific voices over time. The reduction of GM in this area in dyslexic participants could reflect the reduced functional specialization of this region towards the processing of auditory objects, specific to human voices associated with the speech-in-noise deficit observed in dyslexics [13,14].

Another role proposed for the right middle STS involves the processing of speech rhythms and intonation [95]. The processing of intonation primarily relies on variations in pitch-contours (f0), whereas the processing of speech rhythm relies on the processing of slow modulations (envelope). Notably, dyslexic people exhibit deficits in the processing of slow amplitude modulations during auditory and speech processing [96-98] and these processes involve right auditory regions [99]. In addition, this observation is consistent with the hypothesis of an involvement of the right STS in the speech-in-noise perception deficit in dyslexia, as slow modulations are a crucial cue in the processing of speech-in-noise [100]. The question of the causal relation between dyslexia and morphological gray matter plasticity remains. Therefore, additional studies are needed to clarify this issue.

The second major observation in this study was the presence of a significant correlation between the speech-in-noise abilities in the dyslexic group and the WM leftward asymmetry in the posterior STG, indicating that when the WM was more leftward asymmetric in dyslexic individuals, the performance was better in the speech-in-noise test. This result was consistent with a number of studies showing the involvement of the left superior temporal regions in speech processing. Specifically, superior temporal activations in fMRI studies have been obtained when hearing speech-in-noise [101,102]. For example, Zeckveld et al. [102] showed that in difficult listening situations, such as for a -5 dB SNR versus a +20 dB SNR, there was activation in the left posterior STG. We did not observe the same correlation in the normal-readers group, likely reflecting a lack of variability in the results from the speech-in-noise test for this group, as observed in Table 1. This region corresponds to the caudal aspect of BA22, Wernicke’s area, which is a major end of the arcuate fasciculus, its auditory/phonological end. The correlation between white matter density at this location and the speech-in-noise deficit might reflect the phonological/linguistic compound of the speech-in-noise deficit. When listening to speech, this region is often associated with phonological processing and the identification of phonemes in speech streams. When listening to speech in noisy environments, the functional connectivity between these regions and the frontal regions at the other end of the arcuate fasciculus increases [103,104], as motor representations of speech are recruited to facilitate intelligibility. A lack of WM in this region might be associated with difficulties in speech-in-noise comprehension tasks and is ultimately associated with the phonological deficit observed in dyslexia; however, additional studies are needed to further investigate these aspects.

The interpretation of the results of this VBM-based analysis should be carefully undertaken. Indeed, VBM did not provide information on the microscopic structures in the cortical regions, and the results were not specific to underlying tissue properties. The described changes might reflect not only modifications in neuronal density but may also indicate other cellular modifications, such as differences in the density and size of other cell types, myelination or vascularization [36]. Thus, additional studies are required to understand the precise cellular basis underlying dyslexia. In this framework, studies combining the use of different neuroimaging techniques (fMRI, VBM, DTI) and more precise anatomical techniques, such as the measurement of cortical thickness, fiber tracking, or analyses of brain sulci are clearly needed**.**

## Conclusions

The results of this study contribute to the characterization of the morphological deficits in dyslexia. Our major findings confirmed both the presence of a significant deficit in the perception of masked speech and the existence of GM abnormalities in regions traditionally associated with speech processing. Moreover, these results extend the findings of previous studies, showing GM and WM volume reduction in the superior temporal regions and highlighting two important points: first, the rightward GM asymmetry is reduced in the STS, a region involved in processing functions, such as speech-in-noise or auditory temporal processing, which are impaired in dyslexia. Second, speech-in-noise abilities are dependent on the WM asymmetry of the auditory regions, at least in the dyslexic group. These results suggest potentially impaired processing in secondary auditory regions, resulting in difficulties in speech-in-noise comprehension. Taken together, these findings clearly highlight the necessity of functional measures for these regions during speech-in-noise perception to better characterize the involvement of these areas in the deficit present in dyslexia.

## Supporting Information

Figure S1 **Image of the symmetric ROIs used for the analysis.**
(TIF)
