# Supplementary figures and images for: Gray and White Matter Distribution in Dyslexia: A VBM Study of Superior Temporal Gyrus Asymmetry
Source: PLoS One. 2013 Oct 1;8(10):e76823. doi: 10.1371/journal.pone.0076823 (PMC3788100; doi:10.1371/journal.pone.0076823)

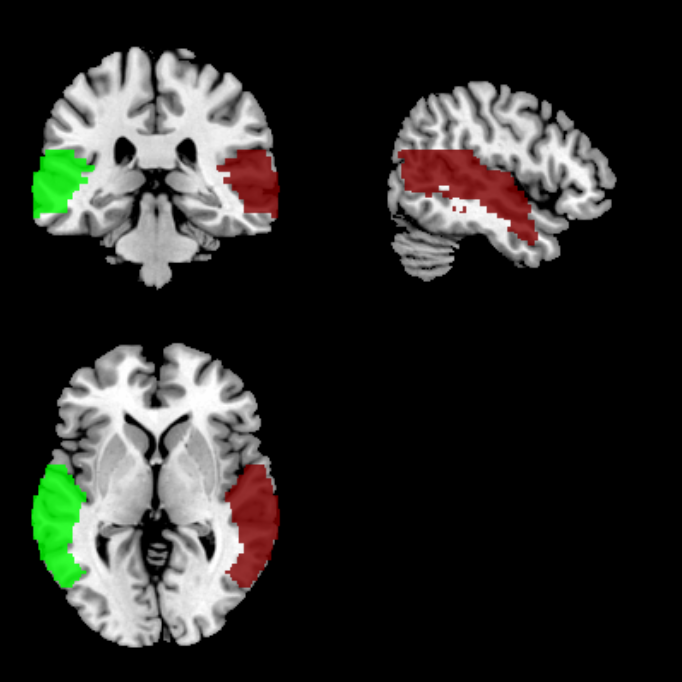

Supplement: Figure S1 — Image of the symmetric ROIs used for the analysis. (TIF) [file pone.0076823.s001.tif]
